# Anti/Vax: Reframing the Vaccination Controversy

**DOI:** 10.5195/jmla.2020.826

**Published:** 2020-01-01

**Authors:** Claire B. Joseph

**Affiliations:** Director, Medical Library, Mount Sinai South Nassau, Oceanside, NY, claire.joseph@snch.org

The title of Bernice L. Hausman’s opinion piece for the *Philadelphia Inquirer,* “Stop Telling Anti-Vaxxers They’re Insane for Questioning Vaccines” [1], offers insight into this excellent and deeply thought-provoking book that delves into the myriad of nuanced reasons why some people distrust and refuse vaccines that modern medicine promotes as safe and public health concerns often require. Hausman, chair of the Department of Humanities at the Penn State University College of Medicine, cogently examines the antivaccine controversy by examining the epistemological, social, and cultural elements that shape the debate.

In the 1980s, 1990s, and early 2000s, anti-vaccination sentiment was fomented in part by reports that some vaccines caused autism and that some included thimerosal, mercury, and other metals. Celebrities like Jenny McCarthy and Robert F. Kennedy Jr. publicly decried the use of vaccines. But “from around 2004,” there emerged the “developing trend of arguing *against* vaccine skeptics and *for* the value of vaccination” (pp. 41–42). It should be noted that “While the overall rate of complete nonvaccination of children remains extremely low (less than 1 percent of the population), there is a perception that nonvaccination is a significant and widespread threat” (p. 32).

Two chapters of Hausman’s text are devoted to nonfiction books on vaccination skepticism written for the general public. Chapter 3, “Whom Do You Trust?,” examines two works that are “pro” vaccination and critical of anti-vaxxers: Paul Offit’s *Deadly Choices: How the Anti-Vaccine Movement Threatens Us All* and Seth Mnookin’s *The Panic Virus: A True Story of Medicine, Science, and Fear.* These works and other writings contributed to the shift in perception of anti-vaxxers as illustrated in a 2015 article in the *New York Times* by Ginia Bellafante. Bellafante’s article referred to a proposed bill in the New York Legislature that would allow parents to opt out of getting their children vaccinated, suggesting that parents would be “permitted to reject vaccinations for their children simply because they opposed them philosophically, as one might oppose Oreos or the Disney Channel” [2].

In contrast to Bellafante and others, chapter 4, “Being a Responsible Parent,” looks at works that explore the multiple and serious reasons why parents refuse or question vaccines for their children: Mark Largent’s *Vaccine: The Debate in Modern America,* Eula Biss’s *On Immunity: An Inoculation,* and Jennifer Reich’s *Calling the Shots: Why Parents Reject Vaccines.*

Other chapters thoughtfully examine “Is Vaccine Refusal a Form of Science Denial” and “What Are Facts, and How Do We Trust Them?” In chapter 9, “Viral Imaginations,” Hausman delves into popular zombie and vampire books and movies that prompt the public imagination regarding the safety of vaccines, including the 2007 film *I Am Legend,* “in which a measles virus engineered for a cancer vaccine makes the entire US population vampiric zombies” (p. 175). It should be noted that this film is based on a 1954 story of the same name by Richard Mathesen, in which a bacterial pandemic creates vampires. Two prior film versions, 1964’s *The Last Man on Earth* and 1971’s *The Omega Man,* both follow Mathesen’s basic premise more closely.

In chapter 10, “Anti/Vax,” Hausman explores interviews about vaccination beliefs conducted by the Vaccination Research Group (VRG) at Virginia Tech over a period of several years and lists twenty reasons why some are suspicious of or resistant to vaccinating their children. Reasons include the desire to avoid unnecessary medicine and treatment, distrust of Big Pharma, distrust of the results of studies of vaccine safety, concerns about the sheer number of vaccines given to children, alternative views about medicine, belief in the value of natural illness, and perception of children having rights over their own bodies.

In her conclusion, “What Vaccination Controversy Can Teach Us about Medicine and Modernity,” Hausman posits that “[e]pistemologically, science does not offer us a lot of help in identifying and explaining the various threads that are woven through vaccination controversy” (p. 220). Instead, Hausman suggests “The answer is science *and something else*” (p. 219). The *something else* requires an objective, nuanced understanding of the many belief and cultural systems that contribute to one’s underlying understanding of and approach to illness and health and how to deal with both. This book is highly recommended.

**Claire B. Joseph, MS, MA, AHIP,** claire.joseph@snch.org, Director, Medical Library, Mount Sinai South Nassau, Oceanside, NY
